# ER Stress Responses: An Emerging Modulator for Innate Immunity

**DOI:** 10.3390/cells9030695

**Published:** 2020-03-12

**Authors:** Giusy Di Conza, Ping-Chih Ho

**Affiliations:** 1 Department of Fundamental Oncology, University of Lausanne, 1007 Lausanne, Switzerland; 2 Ludwig Institute for Cancer Research, University of Lausanne, 1066 Epalinges, Switzerland

**Keywords:** innate immunity, ER stress, infection, chronic diseases

## Abstract

The endoplasmic reticulum (ER) is a critical organelle, storing the majority of calcium and governing protein translation. Thus, it is crucial to keep the homeostasis in all ER components and machineries. The ER stress sensor pathways, including IRE1/sXBP1, PERK/EIf2α and ATF6, orchestrate the major regulatory circuits to ensure ER homeostasis. The embryonic or postnatal lethality that occurs upon genetic depletion of these sensors reveals the essential role of the ER stress pathway in cell biology. In contrast, the impairment or excessive activation of ER stress has been reported to cause or aggravate several diseases such as atherosclerosis, diabetes, NAFDL/NASH, obesity and cancer. Being part of innate immunity, myeloid cells are the first immune cells entering the inflammation site. Upon entry into a metabolically stressed disease environment, activation of ER stress occurs within the myeloid compartment, leading to the modulation of their phenotype and functions. In this review, we discuss causes and consequences of ER stress activation in the myeloid compartment with a special focus on the crosstalk between ER, innate signaling and metabolic environments.

## 1. Introduction

### 1.1. The Endoplasmic Reticulum and its Stress Sensors

The endoplasmic reticulum (ER) is characterized by a peculiar structure whose shape and architecture dictate the biological functions that occurs within this organelle. The ER is composed of a complex system of membranes that gives rise to the NE (nuclear envelope) and peripheral ER, which is composed of sheets and tubules [1]. The synthesis, folding and post-translational modification of secreted and membrane proteins occurs within the sheet, also called rough ER, given the high density of ribosomes loaded with mRNA. On the other side, tubules are smooth and highly curved and host the calcium storage and lipid synthesis machinery. Interestingly, the ER structure is dynamic and can quickly adapt in order to meet cellular demands in response to physiological or pathological stimuli. Similarly, cells with different origins and functions also show diverse architectures of their ERs [2]. Pancreatic beta cells as well as B cells are characterized by enlarged sheet compartments, allowing large production of secreted proteins; however, muscle cells and hepatocytes requiring calcium signaling for contraction and lipid synthesis are distinguished rather by a prevalent tubular network [1].

As guardians of the ER, a three-branch system of proteins acts as a sensor of stress when quality control does not match high standards. This system is known as unfolded protein response (UPR) and undergoes activation upon accumulation of misfolded proteins or excess release of calcium due to leakage of the membranes. The three branches are composed of protein kinase R (PKR)-like endoplasmic reticulum kinase (PERK), inositol-requiring enzyme 1 (IRE1) and activating transcription factor 6 (ATF6) [3]. Normally, all three sensors are kept in their inactive status by binding to the chaperone BiP. BiP actively promotes protein folding, the import of polypeptides and the export of misfolded protein towards the endoplasmic-reticulum-associated protein degradation (ERAD). When misfolded proteins accumulate within the ER lumen, BiP dissociates from PERK, IRE1 and ATF6 and leads to their activation. IRE1 and PERK can therefore switch from monomeric inactive conformations to oligomers that allow autophosphorylation and activation. In addition, ATF6 is cleaved to become an active form and enter the nucleus to promote the transcription of ER-stress responder genes [4]. Interestingly, recent findings have described how PERK and IRE1 can also act as lipid sensors. In the context of an UPR-independent ER stress response, changes in the composition and/or saturation of lipids within the ER membranes can be detected by both PERK and IRE1 through their transmembrane domains [5]. This intriguing discovery has opened new field of investigation and helps us to understand the dynamics of ER stress response in different scenarios.

In mice, genetic knockout of PERK, IRE1 and ATF6 revealed a non-redundant, rather specific role of the three sensors in response not only to pathological but also to physiological ER stress. PERK KO mice are viable but show growth defects associated with skeletal dysplasia, dysfunctional pancreas and hyperglycemia [6]. Pancreatic-specific KO could recapitulate the effect of the global KO, leading to reduced beta cell proliferation, defects in insulin secretion and neonatal diabetes [7]. Given the extraordinary secretory capacity of muscle and pancreatic cells, physiological ER stress occurs during development and explain the phenotype observed in vivo. PERK has a dichotomous role, being able to discern the severity of ER stress and promote growth arrest to allow repair or apoptosis when ER stress is overwhelming. EIf2a is the main target of PERK. Once activated, EIf2a blocks protein synthesis, allowing the cells to resolve the excess of misfolding and restore ER homeostasis. However, in response to prolonged ER stress, PERK/EIf2α induces cell death through the transcription factor CHOP [8,9]. Differently from PERK, IRE1 is not only a kinase but holds the unique ability to promote IRE1-dependent decay (RIDD) of mRNA in order to release a stressed ER from the burden of newcomer proteins [10,11]. In addition to that, IRE1 induces unconventional splicing of its main substrate XBP1 through its RNase domain. Spliced XBP1 mRNA encodes a functional transcription factor that mediates the induction of folding machinery and lipid synthesis genes, both required for a proper ER stress response [12]. Indeed, when dealing with overwhelming protein misfolding, the ER demands high fatty acid and cholesterol generation in order to enlarge its membrane compartment [13]. KO mice of IRE1 and XBP1 are both embryonically lethal [14,15]. However, while the XBP1 KO could be rescued by liver-specific expression of the XBP1 transgene [16], this did not occur in IRE1 KO mice when re-expressing IRE1 in liver [17]. Interestingly, embryonic lethality of IRE1 KO mice, which occurred between day 9 and 11 during development due to vascular defects, was recovered by the expression of endogenous IRE1 in the placenta, beautifully highlighting the importance of physiological ER stress in the extraembryonic tissues and the blood vessel formation in the embryo [17]. These data also suggest that XBP1 and IRE1 play functions that are dissimilar and independent of each other. Finally, ATF6 KO mice also displayed very early embryonic lethality. However, the single knockout mice of the two isoforms, ATF6α and ATF6β, were viable and fertile, suggesting a certain degree of redundancy during embryonic development [18].

The diversity of phenotypes observed upon genetic depletion of IRE1/XBP1, PERK and ATF6 disclose a tissue-specific role but also highlight the possibility that the interaction network of the three branches play an important role to differently activate these pathways upon various stimuli and in different microenvironments. For example, it has been recently shown that in glucose deprived conditions, PERK activates SCAF1 to promote the formation of respiratory supercomplexes within the mitochondria in order to allow alterative energenetic machinery [19]. In contrast, CD4 T cells exposed to low glucose in the tumor microenvironment undergo IRE1/XBP1-mediated ER stress, which reduces mitochondrial respiration by limiting the expression of glutamine carriers on the membrane and therefore blunting glutamine oxidation [20]. These apparently opposite findings suggest that the different ER stress sensor could have a very diverse function in response to similar stimuli, but also highlight the possibility that pathological and physiological environments might preferentially activate one or the other. In this context, it is well known that the ER stress response regulates onset, progression and severity of a variety of pathologies such as cancer, diabetes, atherosclerosis, obesity and neurodegenerative disease, extensively reviewed elsewhere [21,22]. These pathologies have in common some key features, such as infiltration of innate immune cells and metabolic stress. Here we discuss how ER stress is linked to innate immune cells and how metabolism can influence or perturb this balance.

### 1.2. ER Stress and Immune Signaling

Part of the interaction network of ER stress responses contains components of immune signaling pathways, such as STATs, JNK and NFκB. Upon activation, oligomeric IRE1 binds the scaffold protein TRAF2, which is a main component of TNF-receptor signaling [15]. TRAF2 is able to promote TAK1-dependent activation of IKKβ that favors the release of NFκB from its inhibitor IκB [23,24]. In addition to that, TRAF2 is also responsible for ASK1-dependent activation of JNK [25]. PERK can also indirectly induce NFκB through EIf2α. By inhibiting the protein synthesis of IKβ, PERK/EIf2α promote nuclear translocation of NFκB [26]. Both NFκB and JNK are known modulators of inflammation, by promoting the expression of genes and the activity of proteins involved in the regulation of inflammatory response [27]. Among the plethora of cytokines released by the UPR-derived inflammation, IL-6 has been found to be regulated at different levels. In human melanoma cells, spliced XBP1 induces IL-6 expression by directly binding IL-6 promoter. In turn, IL-6 further promotes STAT3 phosphorylation and boosts the pro-oncogenic effect of STAT3 [28]. The link between STAT3 and ER stress has been further strengthened by other evidence. In murine hepatocytes, IRE1 binds and activates STAT3 phosphorylation to foster tissue repair in response to liver damage [29]. Others have shown that in astrocytes, PERK can directly bind JAK1 and promote STAT3 phosphorylation with following increase of IL-6, CCL2 and CCL20 [30]. In the physiological context, the release of proinflammatory cytokines through these pathways functions as damage signals and contributes to the maintenance of tissue homeostasis through induction of cell death when ER stress is overwhelming and cannot be resolved. Interestingly, it has been recently proven that IRE1/XBP1 signaling in myeloid cells can increase pain perception by positively regulating the expression of prostaglandins and eicosanoids [31]. Once again, within a healthy organism this response represents an alert that helps resolution of infection. However, in the pathological context, UPR-dependent activation of prostaglandins or other inflammatory cytokines can rather boost the progression of disease and worsen its outcome.

## 2. ER Stress Response in Myeloid Cells

Myeloid cells are composed of mononuclear and polymorphonuclear cells and, together, take part in innate immunity, representing the essential first line of defense against bacteria, parasites, viruses or endogenous damage. Polymorphonuclear (PMN) cells or granulocytes include neutrophils, eosinophils and basophils/mast cells, while mononuclear cells include macrophages and dendritic cells [32]. PMN cells are characterized by the exceptional presence of granules containing cytotoxic molecules and inflammatory mediators that are specifically secreted upon stimulation by pathogens or other damage [33]. As other secretory cells, PMNs require an extended ER in order to cope with an overload of protein synthesis. On the other side, mononuclear cells are professional antigen-presenting cells (APCs), which also require a perfectly functioning ER to allow antigen processing and presentation [34,35,36].

In the following paragraphs, we review the most recent literature that provides solid evidence of ER stress involvement in the maturation, activation, functions and/or dysfunctions of myeloid cells in different contexts.

### 2.1. Macrophages

ER-stressed macrophages promote, reinforce, swap and in some cases ameliorate disease outcomes. One of the reasons behind the overwhelming presence of ER stress response in macrophages is that both pattern-recognition receptors, Toll-like receptor (TLRs) and nucleotide-binding oligomerization domain-like NOD1 and NOD2 receptors, directly engage IRE1 activation to fully execute a resolutive pro-inflammatory response [37,38]. ER stress inhibitors during *Brucella abortis* infection limit abortion of disease by restraining IL-6 production in an IRE1- and NOD1/NOD2-dependent manner [37]. Similarly, TLR2- and TLR4-dependent splicing of XBP1 occurs upon acute infection with *Francisella tularensis* and essentially contribute to bacteria clearance by directly promoting the production of IL-6 and TNFα [38]. In addition, IRE1 activation in macrophages also promotes inflammasome activation that is essential to foster IL-1b production and to clear bacterial infection [39]. These pioneering discoveries opened the road to a large number of studies that further confirmed the involvement of IRE1/XBP1 in the pathophysiology of macrophage function during acute infection. Many more studies have been then performed in the chronic setting and revealed that persistent ER stress might instead be deleterious, rather than protective, for the progression of diseases. In a model of chronic inflammatory arthritis, myeloid-specific ablation of IRE1 protected the mice from the disease, attenuating the release of pro-inflammatory cytokines [40]. Interestingly, in the same study, the authors mechanistically uncovered that TLR4 promotes TRAF6-dependent ubiquitination of IRE1 that impedes the binding of the phosphatase PP2a, therefore favoring IRE1 phosphorylation [40]. In other chronic diseases, such as obesity and atherosclerosis, ER-stressed macrophages contribute to onset and progression of disease [41,42]. More specifically, it has been shown that in high-fat diet (HFD)-induced obesity, IRE1-depleted macrophages alleviate pathological symptoms by favoring the switch of pro-inflammatory macrophages towards anti-inflammatory [43]. In support of these findings, in a genetic model of HFD-induced NASH (nonalcoholic steatohepatitis) and HCC (hepatocellular carcinoma), treatment with ER stress alleviators PBA and TUDCA, improved disease outcomes by limiting the release of TNF by inflammatory macrophages [44]. TUDCA and 4-PBA are chemical chaperones that work by lowering the burden of unfolded protein within the ER [45]; 4-PBA has also been successfully employed in the treatment of atherosclerosis in mice. In the atherosclerotic plaques, lipid-laden macrophages undergo changes in lipid composition that induce ER stress and engage PERK/CHOP-mediated apoptosis; 4-PBA was able to relieve macrophages from PERK activation, prevent apoptosis and preserve their functions [46]. Accordingly, in a similar model of atherosclerosis, treatment with the IRE1 inhibitors STF083010 or 4μ8c decreased plaque area by reducing macrophage infiltration by limiting IL-1β and the recruiting chemokine CCL2 [47].

**Figure 1:**
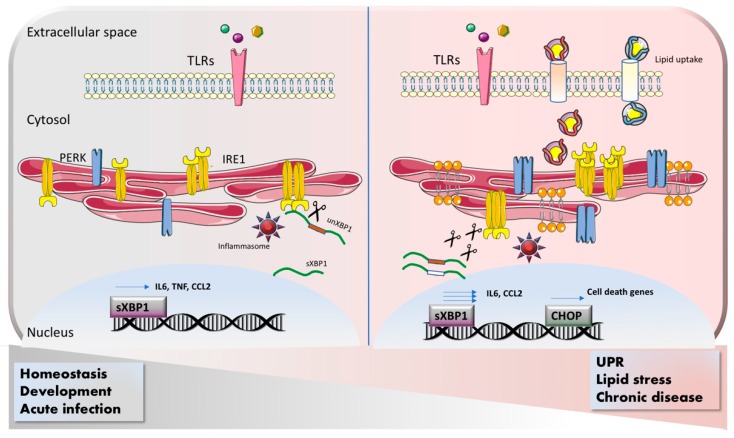
Engagement of ER stress responses in macrophages. Macrophage activation occurs through Toll-like receptors (TLRs) upon binding with DAMPs (Damage-associated molecular patterns) and PAMPs (Pathogen-associated molecular patterns). IRE1, as downstream target of TLRs, induces XBP1 splicing and inflammasome activation therefore promoting generation of pro-inflammatory cytokines. This function of IRE1 is essential to keep homeostasis and to successfully resolve acute infections. On the other hand, many chronic diseases are characterized by a metabolically altered microenvironment, which promotes unfolded protein response (UPR) activation. In addition to that, activated macrophages uptake lipids from the extracellular space, leading to change in the lipid composition of the ER membrane. UPR and lipid accumulation trigger an excessive IRE1/PERK-mediated ER stress response that results in chronic release of pro-inflammatory mediators and induction of cell death that contributes to the progression of disease.

Both atherosclerosis and obesity are mainly characterized by a lipid-enriched microenvironment, and macrophages tend to uptake these metabolites acquiring the status of “foam cell”. Therefore, in these cells, beside UPR or in combination with it, ER stress sensors are mainly activated by changes in the lipid composition and/or saturation of the membranes. It has been recently demonstrated that in adipose-tissue macrophages isolated from obese mice, macrophage-specific depletion of phosphocholine cytidylyltransferase A by limiting the turnover of PC favors the integration of polyunsaturated fatty acid within the ER membrane, therefore reducing ER stress and retaining inflammation [48]. In cancer, tumor-associated macrophages (TAMs) engage the IRE1-dependent ER stress response upon synergistic action of IL-4, IL-6 and IL-10 that promotes cathepsin secretion and increased pro-metastatic phenotype [49]. Interestingly, in this paper the authors found that IL-6/IL-10-dependent STAT3 phosphorylation is upstream of IRE1 activation, adding other level of complexity to the reciprocal regulation of IRE1/STAT3. Beside pro-tumoral macrophage, MDSCs (myeloid-derived suppressive cells) have been recently described as cells expressing both macrophages and granulocyte markers that contribute to the establishment of an immunosuppressive tumor microenvironment, therefore blunting the anti-tumoral immune response [50]. In these cells, tumor-derived stress factors drive CHOP activation in a PERK-dependent manner. By boosting IL6 production, CHOP favors the instauration of an immunosuppressive microenvironment. MDSC-specific deletion of CHOP synergizes with immune checkpoint blockade inhibitors to cure lung, melanoma, thymoma and colon tumors [51]. These findings extensively prove that ER stress is actively involved in pathophysiological mechanisms, having a protective role in acute macrophage response and adverse effects in chronic disease (Figure 1). However, these studies have also opened new fields of investigation that aim to clarify when and how the switch occurs and, most importantly, how the ER stress players are specifically activated in different contexts.

### 2.2. Dendritic Cells

**Figure 2:**
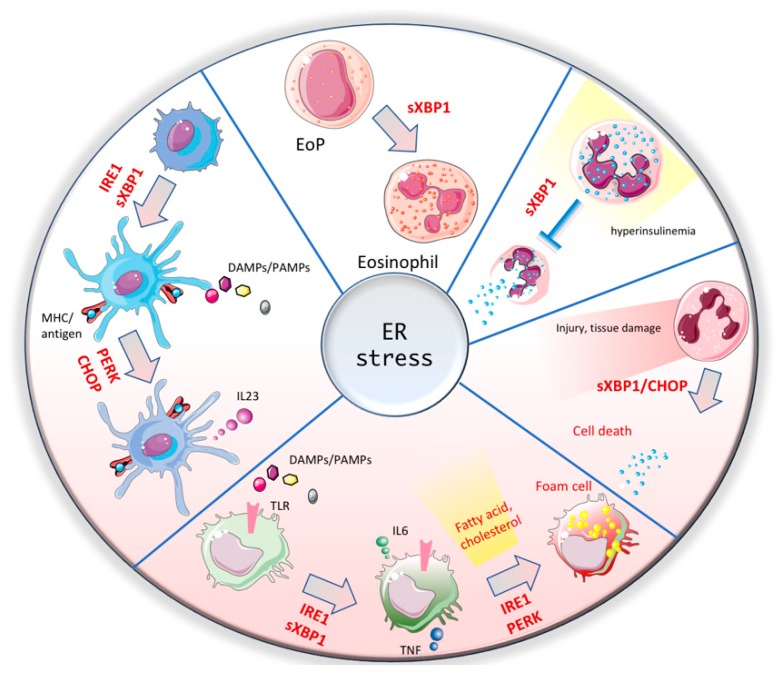
The multifaceted roles of ER stress responses in myeloid cells. ER stress mediators play an important role in modulating development and function of innate immune cells. Immature dendritic cells (in light blue) require IRE1/XBP1 activation in order to differentiate in mature DCs. Mature DCs, upon encountering DAMPs and PAMPs activate PERK/CHOP pathway to accomplish a full activation and the release of IL-23. In macrophages IRE1/XBP1 promotes activation of proinflammatory IL-6 and TNF. However, in disease context, excessive activation of ER stress upon lipid accumulation lead to the formation of foam cells, cell death and impaired resolutive response. In neutrophils, activation of ER stress is associated with increased cell death during development and upon tissue damage. In the context of hyperinsulinemia, mast cells accumulate lipid bodies that cause ER stress response IRE1/XBP1, which results in inhibition of degranulation. Eosinophil precursor (EoP) constitutively activate XBP1 in order to give rise to mature eosinophils.

Dendritic cells (DCs) represent the connection arm between innate and adaptive immunity. DCs are able to cross-present antigens to CD8^+^ and CD4^+^ T cells, unleashing their activation and the engagement of antigen-specific immune responses. Based on location and functions, many subsets of DCs exist, varying from professional antigen-presenting cells to inflammatory DCs. Features and differences of DCs subsets have been nicely reviewed elsewhere [52]. As other innate cells, DCs have myeloid origin and are activated by DAMPs and PAMPs through the expression of PRRs receptors. Once activated, antigen-loaded DCs migrate to draining lymph nodes where they encounter naïve T cells. Binding of the MHC/antigen complex to the T cell receptor in T cells leads to their activation and migration to the site of infection. The molecular mechanism orchestrating the process of antigen presentation is complex and to some extent still not completely uncovered [53]. The ER host the loading of antigens on MHCI or MHCII complexes and for this reason many reports have demonstrated that the ER guardians are indirectly involved in the antigen processing and presentation [54]. In one of the first papers showing the connection between ER stress and DCs, it was drastically proven that XBP1 deletion in the hematopoietic compartment impairs development and survival of dendritic cell lineage [55]. Immature DCs constitutively activate XBP1, which in turn regulates their differentiation in a cell intrinsic manner. However, the molecular mechanism behind this crucial role of XBP1 was not elucidated. Later, more studies clarified that the severity of the phenotype upon genetic loss of XBP1 is different in distinct subsets. Splenic dendritic cells show impaired antigen presentation associated with a disturbed ER architecture. IRE1/XBP1 signaling is constitutively activated in splenic dendritic cells and promotes cross-presentation by directly regulating the expression of proteins crucial for the antigen loading into MHC complex [56]. In contrast, conditional knockout of XBP1 under the CD11c promoter results in ablation of lung DCs via CHOP-mediated apoptosis. In the same mouse model, mucosal DCs survived. However, upon pharmacological blockade of IRE1 activity (which is overactivated in XBP1 KO), mucosal DCs also undergo cell death, suggesting a different threshold of IRE1 activation in different tissues [57]. These studies have been performed in absence of infection and therefore are limited to the physiological status of DCs. However, as it occurs for macrophages, upon TLR activation, ER stress pathways are engaged in DCs and contribute to their function. Strikingly, treatment of DCs with TLR4 and TLR8 agonists induces CHOP activation and its direct binding to the promoter of the pro-inflammatory cytokine IL-23 [58] (Figure 2). Similarly, it has been recently published that palmitic acid or HFD are able to reinforce the effect of TLR7/8 agonist IMQ by boosting IL-23 production in DCs, upon activation of CHOP and sXBP1-mediated ER stress response [59]. In cancer, dendritic cells are loaded with lipid that undergo peroxidation and induce an XBP1-mediated ER stress response. Conditional knockout mice harboring XBP1 deletion in dendritic cells were strongly protected against tumor growth compared to WT mice. Mechanistically, XBP1 boosts triglyceride production and further widens the overloading of lipids, therefore disturbing the machinery of antigen presentation and blunting anti-tumor response [60]. In contrast, a study performed in human graft vs. host disease (GVHD), has shown that DCs-specific sXBP1 deletion strongly suppress alloreactive CD4 T cells, and pharmacological block of spliced XBP1 protects against rejection. Moreover, the authors provided evidence that this phenomenon is restrict to GVHD, because intratumoral T cell response was not affected by XBP1-expressing DCs [61]. In conclusion, by directly or indirectly controlling antigen presentation in combination with TLR signaling, ER stress acts as major hub in regulating DCs functions and therefore represent an attracting therapeutic target. However, more studies are needed in order to fully understand how duration and intensity of inflammation and priming are interplaying with the ER guardians to balance physiologic versus pathologic DC responses.

### 2.3. Granulocytes

#### 2.3.1. Neutrophils

In neutrophils, ER stress sensors have been shown to play an important role to control degranulation process. Indeed, in an acute model of lung injury, activation of complement C5a induces IRE1/XBP1-mediated ER stress, which in turn facilitates degranulation and progression of disease. Neutrophil-specific ablation of XBP1 alleviates tissue injury by decreasing release of granules and proinflammatory cytokines [62]. Others have shown that neutrophil formation requires membrane integrity of the ER and the right ratio of phosphatidylcholine/phosphatidylethanolamine (PC/PE). Genetic inducible knockout of FASN (fatty acid synthase) in bone marrow results in severe neutropenia by specifically reducing the levels of PC within the endoplasmic reticulum of granulocytes, therefore leading to CHOP-mediated ER stress response and massive neutrophil cell death [63]. Similarly, in a model of angiotensin II-mediated cardiac injury, CHOP positively controls neutrophils apoptosis by favoring the resolution of inflammation within the heart [64]. These data suggest that awaking ER stress response during physiological turnover of neutrophils might profoundly affect their representation within the bone marrow. However, upon inflammatory response, CHOP-mediated cell death in neutrophils helps to degranulate and die in order to recruit macrophages to resolve inflammation.

#### 2.3.2. Eosinophils

Eosinophils are at the frontline in the battle against parasites infection and allergies. As other granulocytes, they develop from granulocyte macrophage progenitor (GMP) that give rise to eosinophil progenitor (EoP) [65]. The main evidence that ER stress is involved in eosinophil biology come from a report in which the authors used hematopoietic depletion of XBP1 in order to assess its role in leucocytes development. Strikingly, in these mice eosinophil formation was completely impaired and as compensation more neutrophils or mast cells were produced. The depletion of XBP1 causes cell intrinsic defects by reducing protein folding ability of the cells and favoring the formation of immature granule proteins that are essential for eosinophil formation [66]. As it occurs for other secretory cells, this specific branch of ER stress is activated during development and its role is crucial to keep homeostasis during hematopoiesis.

#### 2.3.3. Basophils/Mast Cells

Basophils and mast cells have many common features but represent two different specific cell type that together with eosinophils, are essential to sustain Th2-immunity in response to helminth infection or allergies. It is technically challenging to work with these cells and for this reason, their functional biology is not well known yet. For the same reason, only few studies have analyzed the role of ER stress response in the pathophysiological function of these cells. In a model of hyperinsulinemia caused by high fat diet, accumulation of lipid bodies in mast cells causes a reshuffling of lipid species within the ER membrane. As consequence, ER stress response is initiated and, differently from what it occurs in neutrophils, it inhibits degranulation [67]. Although the molecular link between ER stress and degranulation has not been fully demonstrated, this report suggests that mast cell dysfunction in insulin resistance might be attributed to ER stress.

## 3. Conclusions

Although extensive research has focused on understanding how ER and stress sensing orchestrate the biology of innate immunity in response to physiological and pathological stimuli (Figure 2), more effort is needed in order to clarify many aspects still unresolved. The role of ATF6 in all myeloid compartments remains largely unexplored due to lack of efficient tools that allow for the proper detection of the activation of this pathway [68]. More insights into ATF6 might help to fill in the dark gaps in the puzzle of the ER stress role in innate immune cells. In addition to that, it became recently clear that the three branches of ER guardians are not only activated by UPR but also by lipid overload and change in the composition of lipid-associated membranes. However, how these changes are sensed is not completely understood yet. It is possible that the stiffness or other physical features of the membranes favors the formation of oligomers and the activation of ER stress pathways. Finally, pharmacological approaches targeting PERK and IRE1 pathways have been initiated and many inhibitors have been employed in various disease contexts in preclinical mouse models or human clinical trials [69]. However, some limitations are defined by the lack of knowledge on different aspects. Genetic studies have highlighted how the block of one branch leads to overcompensation of the others. Careful evaluation of compensatory mechanisms should be evaluated in all disease context. Moreover, as described above, ER stress is necessary for development and physiological function of the innate immune system. Therefore, evaluation of side effects upon exposure to infection or wounds should be carried out in order to assess the risk/benefit balance upon ER stress-targeting therapies.
